# Development and Validation of a Novel Neck Care Module in Patients With Chronic Mechanical Neck Pain

**DOI:** 10.7759/cureus.106473

**Published:** 2026-04-05

**Authors:** Sneha Ganu, Annamma Varghese, Priti Mehendale

**Affiliations:** 1 Musculoskeletal Physiotherapy, K J Somaiya College of Physiotherapy, Mumbai, IND; 2 Physiotherapy, Parul Institute of Doctoral Studies, Parul University, Vadodara, IND; 3 Physiotherapy, K J Somaiya College of Physiotherapy, Mumbai, IND

**Keywords:** content validity, exercises, face validity, mechanical neck pain, patient education, posture correction

## Abstract

Introduction: Neck and back pain represent a major source of disability worldwide and are associated with significant health and economic impacts. Patient education and self-management are recognized as essential components of chronic pain management within multidisciplinary care approaches. However, despite the widespread use of various educational strategies in clinical practice, there is a notable scarcity of literature from India on structured educational materials or programs specifically designed to address chronic neck pain, indicating a critical gap in culturally relevant, evidence-based educational interventions in this context.

Aim and objectives: This study aims to develop and validate a neck care module for patients with chronic mechanical neck pain. The first objective is to develop a structured neck care module specifically designed for individuals experiencing chronic mechanical neck pain. The second objective is to evaluate the developed module for face and content validity to ensure its clarity, relevance, comprehensiveness, and suitability for patient education and clinical use.

Materials and methods: Phase 1 of the study involved the systematic development of a neck care module through four sequential stages. First, a comprehensive literature search was conducted to identify relevant evidence and existing recommendations related to the management of chronic mechanical neck pain. Second, illustrative images were created to visually support the educational content and enhance patient understanding. Third, the module was prepared by integrating evidence-based information with the developed visual materials to ensure clarity and practical applicability. Finally, the preliminary module was reviewed through consultation with multidisciplinary stakeholders involved in pain management to obtain expert input and refine the content. Draft versions of the module were evaluated using the patient education materials assessment tool (PEMAT) to assess understandability and actionability. The final module was validated for content and face validity by experts and the target audience, respectively.

Results: The developed neck care module demonstrated acceptable content validity (CVI = 0.85) and excellent face validity (97.89% agreement), indicating that experts and target users perceive the module’s content as relevant, comprehensive, and appropriate for patients with chronic mechanical pain.

Conclusion: These validation findings support the module’s potential as a reliable educational tool to enhance patient understanding and self-management, warranting further evaluation of its clinical effectiveness in practice.

## Introduction

A considerable health and economic burden is imposed by neck pain, which is one of the leading contributors to the number of years lived with disability worldwide. Epidemiological studies suggest that up to 70% of individuals will experience neck pain at some point in their lifetime, with approximately 50% of those affected experiencing chronic symptoms that persist for more than 12 weeks. Long-term functional limitations and healthcare utilization are significantly influenced by these chronic conditions [1].

The etiology of chronic mechanical neck pain is complex and multifactorial [2]. Factors that may contribute to the etiology of chronic mechanical neck pain include prolonged sedentary behavior, poor postural habits, reduced muscle strength in the cervical region, and abnormal cervical movement patterns. Contemporary management strategies have, over time, evolved from unimodal to multimodal evidence-based strategies with the main goal of optimizing patient outcomes with minimum risks and costs involved [3]. Clinical practice guidelines have recommended a variety of conservative management strategies like patient counseling and education, tailormade exercise programs, manual therapy techniques, and proper administration of pharmacological agents, to be used in the management of acute, subacute, and chronic neck pain disorders. Patient education plays a major role in pain amelioration and coping mechanisms that could greatly improve the efficacy outcomes and thus lead to a decrement in health care expenditures. Technology-based methods like telehealth have been used in the rehabilitation of patients with disorders like stroke, post-orthopedic surgery, and psychological disorders, which could be effectively used in pain disorders like musculoskeletal pain disorders [4,5]. However, it has not been widely used in pain disorders like musculoskeletal pain disorders due to the perception that passive treatment techniques have to be used in pain disorders like musculoskeletal pain disorders. However, evidence has shown that passive treatment techniques are not necessary in pain disorders like neck pain disorders [6]. Rather, active techniques like self-efficacy techniques should be used in innovative ways rather than face-to-face sessions. Different types of patient education are frequently used in clinical settings. Patient education can include both written and verbal forms of education and can be both a separate entity and a part of an intervention program. Patient education can also be carried out for a single patient or for groups of patients. Patient educational booklets are most frequently used for carrying out written patient education. Patient educational booklets are designed to carry out evidence-based education in an easy-to-understand manner for the patient. Evidence indicates a notable improvement in chronic low back pain (CLBP) using an educational booklet [7,8]. However, a scarcity of literature is present in neck pain patients. Hence, the aim of this study was to develop and validate a new form of self-instructional neck care module consisting of educational content on pain neuroscience, ergonomic guidance, self-management strategies, etc., for use in carrying out educational and rehabilitative programs in neck pain management.

## Materials and methods

The study was approved by the Institutional Ethics Committee, K J Somaiya Medical College, Sion, Mumbai, and CTRI registration was done. The study was carried out in two phases: development of the neck care module and validation of the developed module.

Phase 1

The neck care module was created in a manner that provides updated evidence-based information in an easily understandable form for patients suffering from neck pain. The process of developing the module was done in several phases (i) literature search, (ii) creation of pictorial forms of postures and exercises, (iii) creation of the module containing evidence-based information along with appropriate illustrations, and (iv) seeking inputs from various stakeholders in pain management practices, i.e., physiotherapists, orthopedics, physicians, and yoga experts. The key components of the module were introduction to spine, causes and risk factors for neck pain, do's and dont's to be followed, guidance on seeking help of a consultant when needed, simple exercises, etc. The module was created in simple English. The drafts of the module were evaluated using a tool called the patient education materials assessment tool (PEMAT), which is an Instrument To Assess the Understandability and Actionability of Print and Audiovisual Patient Education Materials (Version 1.0) [9,10]. PEMAT-P for printable materials (e.g., brochures, pamphlets, PDFs) is a 17-item tool for measuring understandability, and a 7-item tool for measuring actionability was used for evaluation of the neck care module by five physiotherapy professionals. It was found that the module had good quality, as evidenced by satisfactory scores for understandability and actionability.

Phase 2

The developed module on neck care was shared among the key stakeholders, such as physiotherapists, orthopedists, yoga experts, and the target audience, for assessing the content validity and face validity, and accordingly, the module was modified on the basis of the feedback and suggestions received from the stakeholders. For the validation of the booklet, two measures that are recommended by Castro et al. for the development and validation of printed educational materials were used. [11] Content validity and face validity were conducted by experts and the target audience, respectively. 

The statistical analysis of content validity was performed to quantify the level of agreement among experts regarding the relevance of items included in the neck care module. Expert ratings were obtained using a 4-point Likert scale, where scores of 3 (quite relevant) and 4 (highly relevant) were considered indicative of relevance. The Item-Level Content Validity Index (I-CVI) was calculated for each item as the proportion of experts assigning a rating of 3 or 4. Items with I-CVI values ≥0.78 were considered acceptable, while those below this threshold were subjected to revision or removal. The overall content validity of the module was assessed using the Scale-Level Content Validity Index (S-CVI), computed both as the universal agreement among experts (S-CVI/UA) and as the average of I-CVI values across all items (S-CVI/Ave), with values ≥0.80 indicating excellent content validity. 

Face validity of the neck care module was assessed using a descriptive methodological approach based on the level of agreement among participants. A purposive sample of 10 patients with chronic mechanical neck pain was recruited. Participants were asked to review the module and rate its clarity, comprehensibility, language, layout, relevance, and usefulness using a 5-point Likert scale ranging from strongly disagree (1) to strongly agree (5). The level of agreement was calculated as the percentage of participants who rated each item as “agree” or “strongly agree.” A level of agreement of ≥80% was considered indicative of acceptable face validity, while items with lower agreement were revised. Descriptive statistics, including mean and standard deviation, were also computed. Qualitative feedback was collected to identify areas for improvement, and necessary modifications were made to enhance the overall usability and clarity of the module.

The developed and validated Neck Care Module has been officially copyrighted by the Copyright Office, Government of Maharashtra (Registration No. L-154999/2024, dated 20 April 2024).

## Results

As shown in Table 1, the content validity of the neck care module was established by evaluating it with a panel of 10 experts. The experts had a mean (SD) of 16.9 (5.11) years of professional experience and a mean (SD) of 39.6 (8.76) years of age, showing a well-experienced and representative sample of experts. Content validity in terms of I-CVI and S-CVI was calculated for relevance and content. The I-CVI of all items for relevance was between 0.8 and 1, which falls within the acceptable CVI range. The S-CVI/Ave for relevance was 0.85, and this falls within acceptable scores, showing good content validity and thus supporting the scientific accuracy, relevance, and appropriateness of the neck care module for patient education purposes.

**Table 1:** Face Validity

Component	Level of Agreement
Literary Presentation	92.14%
Illustrations	99.33%
Material is sufficiently specific and understandable	98%
Legibility and Printing Characteristics	100%
Quality of Information	100%
Face validity	97.89%

As shown in Table 2, the face validity of the neck care module was examined by 10 patients with neck pain, of whom five were males and five females, with a mean age of 42.7 ± 4.2 years. Face validity in terms of I-FVI and S-FVI was calculated.

**Table 2:** Content Validity

Name	Value
No of experts	10
Years of experience	Mean(SD): 16.9 (5.11)
Age	Mean(SD): 39.6 (8.76)
CVI Scientific Accuracy	0.8
a) contents are in agreement with the current knowledge	0.8
b) recommendations are necessary and are correctly approached	0.8
CVI Content	0.9
a) objectives are evident	0.8
b) recommendation about the desired behavior is satisfactory	0.8
c) there is no unnecessary information	1
d) important points are reviewed	1
Overall Content Validity	0.85

Excellent acceptability and clarity of the material were established by the high level of agreement in all the domains that were examined. The illustrations scored an excellent score of 99.33%; the literary presentation scored a consensus level of 92.14%; the material was accepted as sufficiently precise and understandable by almost all the participants (98%); the quality of the information, the legibility, and the printing characteristics were all agreed upon at 100%. The material was well-designed, easy to read and understand, and appropriate from the point of view of the population in question, as indicated by the 97.89% face validity of the neck care module.

## Discussion

The present study aimed to develop and validate a neck care module for patients with chronic mechanical neck pain. Content validity is a fundamental component in the development of educational and clinical tools. It determines the extent to which the content adequately represents the construct it intends to measure. The findings of this study indicate that the neck care module demonstrates good content validity, supporting its scientific accuracy, relevance, and appropriateness for patient education. The Content Validity Index for scientific accuracy was 0.80, which meets the minimum acceptable criterion for content validity given by Polit and Beck [12]. This suggests that the module content is largely evidence-based and scientifically sound. While slightly lower than the CVI values for other domains, this score still indicates acceptable agreement among experts and points to the robustness of the scientific foundation of the module. As per the methodology suggested by Grant & Davis, minor revisions based on expert feedback may further enhance scientific precision, which is a recommended practice in tool development [13]. The CVI for content relevance and adequacy was higher, i.e., 0.90. This is because expert opinion was high on the content of the module being relevant and adequate. Relevance and adequacy are critical for patient education materials because they affect patient understanding and adherence to recommended practices [14]. High scores for content relevance indicate that the module covers critical issues in neck care that are relevant in a real-world setting. The overall content validity score of 0.85 was found to indicate good content validity, since Polit and Beck (2006) noted that content validity was acceptable for newly developed educational materials, especially when the score was higher than 0.80. This supported the neck care module’s suitability for patient education practice.

Face validity is an essential preliminary step in the evaluation of patient education material, as it measures the acceptability, understandability, and usefulness of the material to the target population, as stated by Polit and Beck [15]. In this study, face validity of the neck care module was carried out by patients with neck pain, and this ensured that feedback on the material was obtained directly from the target population. Inclusion of members of the target demographic within the study is an essential part of face validity, as it can be used to determine the material’s relevance to its intended use [16]. The high levels of agreement found across all domains suggest that the material was well received by the patients, meeting their information needs. This study has clearly highlighted that the material has excellent face validity, making it suitable for patient education. The literary presentation scored a high level of agreement, 92.14%, which indicates that the language, structure, and organization of the content were appropriate for patient understanding. The literary presentation of health education content is known to improve patient understanding of health information, which is essential for patient management and compliance with recommended care practices [16]. The extremely high rating for illustrations, 99.33%, indicates the importance of illustrations as tools for patient education. The use of illustrations is crucial for improving patient understanding of health information, which is often complex, especially for patients with different levels of health literacy [17].

The majority of participants agreed (98%) on the point that the information was specific and easy to understand, thus implying that the information was not too technical or too vague. This balance of information is crucial in patient education, since information that is too technical could lead to confusion, and on the other hand, information that is too vague could fail to address the concerns of the patient [18]. The unanimous agreement on legibility, printing characteristics, and the quality of information (100%) also speaks to the usability of the information in the module. Font, layout, and printing style have been cited to have a significant effect on patient engagement in patient education materials. Face validity is critical since it is a fundamental prerequisite for successful implementation of any educational tool by patients [18]. The high face validity percentage of 97.89% indicates that the design of the neck care module was appropriate and easy to understand by patients with neck pain.

These results, together with content validity, confirm that the neck care module can be an effective tool for patient education.

Future scope

Future phases of this study will evaluate the clinical effectiveness of the neck care module on patient outcomes such as pain, function, and quality of life. Further work may include language translations and cultural adaptations to enhance accessibility and applicability across diverse populations. These steps aim to support wider implementation in clinical practice and self-management programs.

Limitations

Since the neck care module was only created in English, people who don't speak the language well may find it difficult to use it. For broader applicability, translation and validation in regional languages are advised.

## Conclusions

These validation results show that, according to expert evaluation, the developed neck care module exhibits acceptable levels of content relevance, clarity, and consistency. This implies that patients with chronic mechanical neck pain may find the module to be a reliable educational tool. The module has the potential to increase patients' understanding and awareness of their condition by offering structured information about the condition, proper neck care techniques, posture correction, and self-management strategies.
